# Supplemental parenteral nutrition in critically ill patients: a study protocol for a phase II randomised controlled trial

**DOI:** 10.1186/s13063-015-1118-y

**Published:** 2015-12-24

**Authors:** Emma J. Ridley, Andrew R. Davies, Rachael Parke, Michael Bailey, Colin McArthur, Lyn Gillanders, David J. Cooper, Shay McGuinness, For the Supplemental Parenteral Nutrition Clinical Investigators

**Affiliations:** Australian and New Zealand Intensive Care Research Centre, School of Public Health and Preventative Medicine, Monash University, Commercial Road, Melbourne, 3004 Australia; Nutrition Department, Alfred Health, Commercial Road, Melbourne, 3004 Australia; Cardiothoracic and Vascular Intensive Care Unit, Auckland City Hospital, Park Road, Grafton, Auckland New Zealand; Intensive Care Unit, The Alfred Hospital, Commercial Road, Melbourne, 3004 Australia; Medical Research Institute of New Zealand, Wellington, New Zealand; Faculty of Medical and Health Sciences, University of Auckland, Park Road, Grafton, Auckland New Zealand; The Department of Critical Care Medicine, Auckland City Hospital, Park Road, Grafton, Auckland New Zealand; Nutrition and Dietetics, Auckland City Hospital, Park Road, Grafton, Auckland New Zealand

**Keywords:** Clinical nutrition, Nutrition therapy, Enteral nutrition, Parenteral nutrition, Critical care, Randomised controlled trials

## Abstract

**Background:**

Nutrition is one of the fundamentals of care provided to critically ill adults. The volume of enteral nutrition received, however, is often much less than prescribed due to multiple functional and process issues. To deliver the prescribed volume and correct the energy deficit associated with enteral nutrition alone, parenteral nutrition can be used in combination (termed “supplemental parenteral nutrition”), but benefits of this method have not been firmly established. A multi-centre, randomised, clinical trial is currently underway to determine if prescribed energy requirements can be provided to critically ill patients by using a supplemental parenteral nutrition strategy in the critically ill.

**Methods/design:**

This prospective, multi-centre, randomised, stratified, parallel-group, controlled, phase II trial aims to determine whether a supplemental parenteral nutrition strategy will reliably and safely increase energy intake when compared to usual care. The study will be conducted for 100 critically ill adults with at least one organ system failure and evidence of insufficient enteral intake from six intensive care units in Australia and New Zealand. Enrolled patients will be allocated to either a supplemental parenteral nutrition strategy for 7 days post randomisation or to usual care with enteral nutrition. The primary outcome will be the average energy amount delivered from nutrition therapy over the first 7 days of the study period. Secondary outcomes include protein delivery for 7 days post randomisation; total energy and protein delivery, antibiotic use and organ failure rates (up to 28 days); duration of ventilation, length of intensive care unit and hospital stay. At both intensive care unit and hospital discharge strength and health-related quality of life assessments will be undertaken. Study participants will be followed up for health-related quality of life, resource utilisation and survival at 90 and 180 days post randomisation (unless death occurs first).

**Discussion:**

This trial aims to determine if provision of a supplemental parenteral nutrition strategy to critically ill adults will increase energy intake compared to usual care in Australia and New Zealand. Trial outcomes will guide development of a subsequent larger randomised controlled trial.

**Trial registration:**

NCT01847534 (First registered 5 February 2013, last updated 14 October 2015)

**Electronic supplementary material:**

The online version of this article (doi:10.1186/s13063-015-1118-y) contains supplementary material, which is available to authorized users.

## Background

In critical illness, enteral nutrition (EN) is usually delivered to provide estimated daily nutrition requirements via a gastric tube [1–3]. EN is the preferred choice of nutrition for critically ill adults because it mimics normal nutritional intake in health, preserves gastrointestinal tract (GIT) function, is relatively inexpensive and has been associated with a reduced incidence of pneumonia and mortality when started early after intensive care unit (ICU) admission [4–6]. The alternative to EN is parenteral nutrition (PN), which is a specialised solution designed to provide daily nutrition requirements intravenously. PN is used when a patient does not have a functioning GIT or when a clinical preference for use of PN exists [1, 7]. Until recently it was thought that PN was associated with an increased risk of infectious complications and mortality, although new data indicates that these risks may have reduced with contemporary care in the ICU [8, 9].

It has been reported that only 45–60 % [10] of energy is provided when EN is used alone due to delivery and tolerance problems [11], resulting in failure to meet daily energy requirements with unknown consequences. The strategy of “supplemental PN” aims to correct the energy deficit from inadequately delivered EN with a supply of PN, to meet 100 % of daily energy requirements in combination. This approach is based on the premise that delivery of close to 100 % of estimated daily nutrition requirements may improve patient outcomes. Whilst the strategy has been demonstrated to deliver close to 100 % of estimated energy needs, the effects on clinical outcomes have been contradictory [12–14]. A prospective randomised control trial (RCT) which investigated supplemental PN initiated early (within 48 hours of ICU admission) versus late (8 days after ICU admission) demonstrated that late supplemental PN resulted in patients being more likely to be discharged earlier from the ICU, with fewer infections when compared to patients in the early arm. However, late supplemental PN led to a higher proportion of hypoglycaemia, a more pronounced inflammatory response and did not affect overall hospital, 90-day mortality or functional status [15]. The outcomes from this study appear to be contradictory and may relate to the use of aggressive insulin therapy, which is not practiced in Australia and New Zealand (ANZ) [16]. Furthermore, the population in this RCT were largely patients undergoing cardiac surgery, and of low to moderate acuity. This patient group can usually return to volitional oral intake quickly and do not often require artificial nutrition due to their short duration of ICU stay; thus, it would seem there may be a low likelihood of benefit from supplemental PN in this population. Another RCT investigating supplemental PN from admission to ICU versus usual care found that the supplemental PN group received more energy (28 kcal/kg per day versus 20 kcal/kg per day) and had fewer nosocomial infections compared with the usual care group (27 % versus 38 %, respectively), but only on days 9–28 of ICU admission [14]. This finding may be explained by the positive effect of adequately delivered nutrition on immunity later in the ICU stay, which is also a biologically plausible explanation.

Thus, it seems that supplemental PN in addition to standard EN may be able to deliver increased energy to critically ill adults, but the exact clinical effects and the population that may benefit most remain undefined. Our aim is to determine if a supplemental PN strategy commenced 48–72 hours following ICU admission will deliver increased amounts of energy to adults with severe critical illness, when compared with usual care in six ANZ tertiary ICUs.

## Methods

### Design and study participants

A stratified, prospective, multi-centre, unblinded, randomised, parallel-group phase II study will be undertaken.

### Inclusion criteria

1. Admitted to intensive care between 48 hours and 72 hours previously
2. Mechanically ventilated at the time of enrolment and expected to remain ventilated until the day after tomorrow
3. At least 16 years of age
4. Have central venous access suitable for PN solution administration
5. Have one or more organ system failure related to their acute illness defined as:

- PaO_2_/FiO_2_ ≤ 300 mmHg
- Currently on one or more continuous vasopressor infusions which were started at least 4 hours ago at a minimum dose of:

- Dopamine ≥ 5 mcg/kg/min
- Noradrenaline ≥ 0.1 mcg/kg/min
- Adrenaline ≥ 0.1 mcg/kg/min
- Any dose of vasopressin
- Milrinone > 0.25 mcg/kg/min)

6) Renal dysfunction defined as

In patients without known renal disease:

- Serum creatinine > 171 mmol/L OR
- Currently receiving renal replacement therapy

In patients with known renal disease:

- An absolute increase of > 50 % in serum creatinine from baseline OR
- Currently receiving renal replacement therapy

7) Currently has an intracranial pressure monitor or ventricular drain in situ
8) Currently receiving extracorporeal membrane oxygenation
9) Currently has a ventricular assist device.

### Exclusion criteria

Patients will be excluded if:

1. Both EN and PN cannot be delivered at enrolment (that is, either an enteral tube or a central venous catheter cannot be placed or clinicians feel that EN or PN cannot be safely administered due to any other reason)
2. Currently receiving PN
3. Standard PN solutions cannot be delivered at enrolment (that is, clinicians believe that a patient definitely needs a specific parenteral nutrition formulation (for example, glutamine supplementation or specific lipid formulation)
4. Death is imminent or deemed highly likely in the next 96 hours
5. There is a current treatment limitation in place or the patient is unlikely to survive to 6 months due to underlying illness
6. More than 80 % of energy requirements have been satisfactorily delivered via the enteral route in the last 24 hours
7. Are known to be pregnant
8. The treating clinician does not believe the study to be in the best interest of the patient.

### Randomisation, allocation concealment and blinding

Concealed randomisation will be performed via a web-based system which includes randomisation in blocks of 6 at each site. Treatment allocation will be stratified by site. The trial is unblinded.

### Trial intervention and comparator

The intervention is the delivery of a supplemental PN strategy using Olimel N9-840E/Triomel 9, manufactured and supplied by Baxter Healthcare Corporation, Old Toongabbie NSW 2146, Australia. A multi-trace element solution (10 ml), multi-vitamin (Cernevit, Baxter Healthcare Corporation, 5 ml) and ascorbate (300 milligrams) for stability will be added to the intervention in a Baxter Healthcare Corporation compounding centre following good manufacturing practice.

Further details on the interventional product can be viewed at Additional file 1.

The comparator arm will be usual care, with provision and management of nutrition as per local practice at each participating site.

The intervention period is defined as 7 days from the day of randomisation.

### Study procedures common to both arms

Patients will be screened for eligibility by research coordinators/medical staff at each site when they are between 48 and 72 hours of their first admission to the ICU. Those that are found to meet all the inclusion and none of the exclusion criteria will be randomised using a web-based randomisation system.

At randomisation, the body weight of study participants will be standardised using calculated body weight (CBW). To determine CBW, actual or estimated weight and height will be required to allow calculation of body mass index (BMI). The weight used to determine BMI will be defined according to the following hierarchy:

- Actual body weight if it has been recorded in the previous 6 weeks
- Estimated dry weight if actual weight is not known.

Height will be estimated using demi arm span [17].

CBW will be the patient’s actual weight if their BMI is deemed to be <25 kg/m^2^. If their BMI is ≥25 kg/m^2^, the CBW will be set to the patient’s ideal weight at a BMI of 23 kg/m^2^. Once the CBW has been determined, it will not be changed for the study duration.

Daily energy requirements will be estimated using CBW with a fixed prescription. The daily energy requirements will be set at 25 kcal/kg CBW unless the patient is receiving renal replacement therapy (RRT) and/or extracorporeal membrane therapy (ECMO), where 30 kcal/kg of CBW will be used. The daily energy requirement will only be changed during the study period if the patient commences or discontinues ECMO and/or RRT (with the two requirement options being 30 kcal/kg CBW or 25 kcal/kg CBW, respectively). A higher energy requirement has been chosen during RRT and/or ECMO due to the potential for increased metabolic stress and inflammation associated with the delivery of both therapies and the underlying disease processes that require these treatments [18]. Once randomised, the target rate for continuous EN delivery will be calculated by the treating clinical team to match the daily energy requirement, with the assumption that all patients should receive 100 % of their daily energy requirements from administration of EN and rounded up to the nearest 5 ml/hour. The choice of EN formula, protein requirement estimation and management of blood glucose levels will be according to local protocols.

**Fig. 1:**
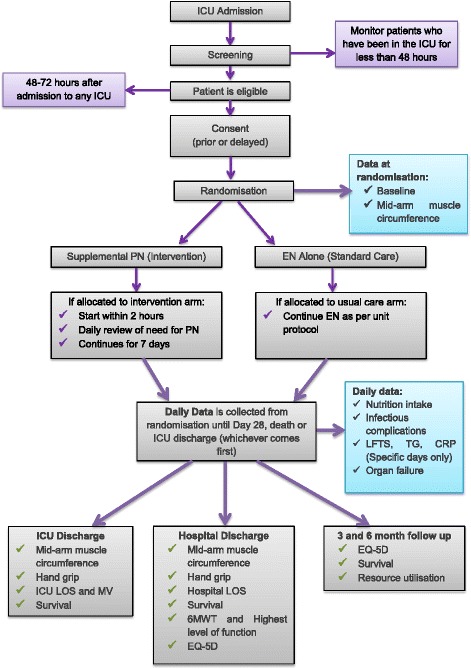
Study overview. CRP: C-reactive protein; EN: enteral nutrition; EQ-5D: EuroQuol 5 dimension; ICU: intensive care unit; LFTs: liver function tests; LOS: length of stay; MV: mechanical ventilation; PN: parenteral nutrition; 6MWT: 6-minute walk test

Figure 1 demonstrates the study processes from screening to study completion.

### Study procedures in the intervention arm

Day of randomisation:

- The interventional product will be administered to intervention patients within 2 hours of randomisation via a central venous catheter (including long-term central catheters, for example, a Hickman catheter if already in situ) or a peripherally inserted central catheter. Management of the line will be as per the participating hospital’s usual procedure. Due to the increased risk of overfeeding with energy when PN is used, the intervention strategy has been designed to minimise this risk. Thus, the maximum amount of energy provided by the intervention will be 20 kcal/kg/day (or 24 kcal/kg/day for those on RRT and/or ECMO), which equals 80 % of the daily energy requirement set at 25 or 30 kcal/kg/day, respectively. This will allow for small amounts of energy provided by EN, 25/50 % glucose and propofol (non-nutritional energy sources) in addition to interventional product in the intervention arm.

The starting rate of PN will be determined by the amount of energy received via the enteral route in the 24 hours prior to randomisation:

- Between 40–80 % of daily energy requirement received from EN: PN rate will equal delivery of 10 kcal/kg of CBW/day (or 12 kcal/kg of CBW/day for those on RRT and/or ECMO)
- Less than 40 % of daily energy requirement received from EN: PN rate will equal delivery of 20 kcal/kg of CBW/day (or 24 kcal/kg of CBW/day for those on RRT and/or ECMO).

Management of EN in the intervention arm will be according to unit protocol. Every attempt will be made by the treating clinical team to achieve delivery of EN in the intervention arm to provide 100 % of daily energy requirements. Importantly, EN must not be reduced based on the amount of intervention being administered.

**Fig. 2:**
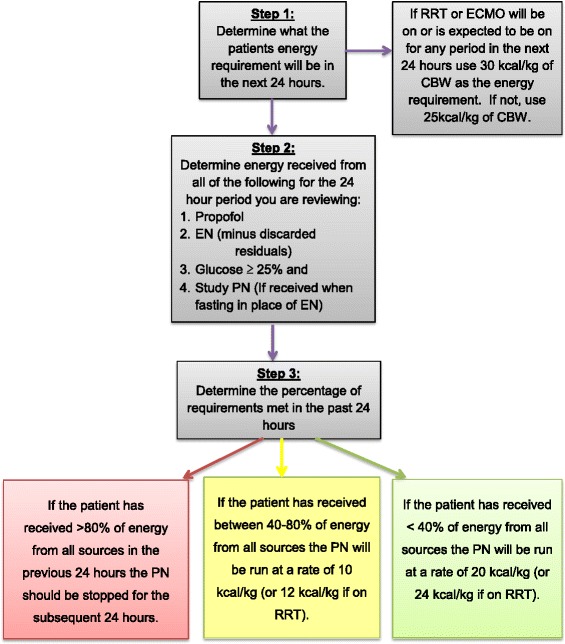
Daily adjustment of PN rate in intervention arm. ECMO: extracorporeal membrane oxygenation; EN: enteral nutrition; PN: parenteral nutrition; RRT: renal replacement therapy

Daily review of intervention:

- From study day 2 until study day 7 (or ICU discharge, whichever occurs first), the adequacy of energy from EN and non-nutritional sources will be assessed at midday by a member of the site research team. Total energy intake will be determined for the 24 hours prior to review and used to determine the rate of delivery of study PN for the subsequent 24 hours (Fig. 2). Once the rate is set for the following 24 hours by the research team, it should not be altered by the treating team unless deemed necessary for patient safety.

Management of interruptions to EN in the intervention arm:

- In the event of an anticipated or actual interruption to EN for a period of 2 hours or more in the intervention arm, the interventional strategy will be adjusted to minimise energy deficit for the period of the interruption. During the interruption period, the intervention will be run at the hourly rate corresponding to 20 kcal/kg or 24 kcal/kg for those on RRT and/or ECMO. If the patient is already receiving the highest rate of the intervention, there will be no change to the rate during the interruption period. As soon as is practical, EN should be recommenced as per local protocol and the intervention returned to the rate determined as per the midday assessment.

Cessation of study intervention prior to the end of the study period:

- The intervention will cease either prior to ICU discharge or 7 days following enrolment if energy from EN and non-nutritional sources provides more than 80 % of estimated energy requirements on any day. Cessation on any one day will not preclude recommencement in the following 24 hours should the strategy be indicated based on the procedures previously outlined, until study day 7.
- Should a patient commence oral intake during the 7-day study period, the intervention will cease when it is deemed that the patient will resume oral intake with the intent to provide nutrition, that is, not only to provide water or fluid intake.

### Usual care arm

After enrolment, patients allocated to the usual care arm will commence or continue EN via an enteral tube to a target rate aimed to provide 100 % of daily energy requirements. All other aspects of nutrition therapy will be managed according to local unit protocol and, if required, include the use of promotility agents and the placement of nasojejunal feeding tubes prior to commencement of PN. PN will only be used in the usual care arm if the above methods have been attempted, or if an absolute contraindication to EN develops. The interventional product will be used in the usual care arm should PN be required within 7 days of randomisation. If PN is required after study day 7, it will be the usual hospital PN formula, managed by the treating clinicians as clinically appropriate.

### Outcome measures

The primary outcome of this trial is the mean energy amount in calories delivered from nutrition therapy over the first 7 days of the study period.

Secondary outcomes include:

1. Total protein amount delivered in the first 7 days of the study period
2. Total energy amount delivered in the ICU stay (up to 28 days)
3. Total protein amount delivered in the ICU stay (up to 28 days)
4. Total antibiotic usage
5. Sequential Organ Failure Assessment scores
6. Duration of mechanical ventilation
7. Duration of ICU and hospital stay
8. Mortality to 180 days post randomisation
9. Functional and quality of life to 180 days post randomisation

### Study management and data collection

This trial will be coordinated by the Australian and New Zealand Intensive Care Research Centre (ANZIC-RC), Monash University, Melbourne, Australia. Dedicated study tools will be provided to participating sites to standardise all study procedures. Data will be collected at each site by dedicated and trained research staff using a paper case report form. Study variables collected will include baseline demographics such as anthropometric measurements, admission diagnoses, physiological parameters, Acute Physiology and Chromic Health Evaluation II, daily information including nutrition therapy, antibiotic use, blood tests and outcome data such as mortality, protocol deviations and serious adverse events (SAEs). At ICU and hospital discharge, functional, strength and health-related quality of life (HRQOL) assessments will be undertaken using the 6-minute walk test if possible and/or the highest level of function scale [19], hand grip strength and the EuroQol 5 dimension 5 level (EQ-5D-5 L) tools, respectively. Study participants will be contacted at 90 and 180 days post randomisation (unless previously deceased) to assess HRQOL, resource utilisation and survival. Follow-up assessments will be conducted via telephone by the research staff at the randomising site using a pre-prepared script to obtain the assessment using the EQ-5D-5 L. In the event that the patient is unable to complete the assessment at any time point, a relative or friend for the patient will be used as per the instructions for the EQ-5D-5 L. Data will be entered by the research staff at each participating site into a web-based database developed by Spiral Web Solutions, Wellington, New Zealand. Table 1 details the full table of events from baseline to outcome assessment.

**Table 1:** Table of events: usual care and intervention arms

Study day	Baseline	1	2	3	4	5	6	7	8	9	10	11	12	13	14	21	28	ICU D/C	Ward	Hospital D/C	90 days post D/C	180 days post D/C
Incl. and excl. criteria	X																					
Consent	X																					
Randomisation	X																					
Demographics	X																					
Apache II score	X																					
Apache III diagnosis	X																					
Daily data^a^ (ICU)	X	X	X	X	X	X	X	X	X	X	X	X	X	X	X	X	X					
LFTs, WBC	X	x	x	x	x	x	x	X	x	x	x	x	x	x	X	X	X					
Use of new antibiotics	X	X	X	X	X	X	X	X	X	X	X	X	X	X	X	X	X					
SOFA score	X	X	X	X				X							X	X	X					
TG	X			X				X							X							
CRP	X							X							X		X					
Dur MV																		X				
LOS ICU																		X				
LOS hospital																				X		
Survival status																		X		X	X	X
Mid-upper arm muscle circumference	X									Measured once patient is ready for ICU D/C										X		
Hand grip										Measured once patient is ready for ICU D/C										X		
6-minute walk test																				X		
QOL																				X	X	X

X denotes must be collected on specified day

x denotes collect only if measured, no need to specially collect

Abbreviations used in table: *CRP* C-reactive protein, *EN* enteral nutrition, *ICU* intensive care unit, *LOS* length of stay, *MV* mechanical ventilation, *PN* parenteral nutrition, *QOL* quality of life, *SOFA* Sequential Organ Failure Assessment, *TG* triglycerides

^a^Daily data: The following variables will be collected daily: target energy and protein requirements, received energy and protein amounts, received EN and PN volumes, AM BGL levels, units of insulin delivered, gastric residual volumes, documented episodes of vomiting, documented episode of abdominal distension, documented episode of witnessed aspiration

### Ethical considerations

The study protocol has been approved by The Alfred Hospital Ethics Committee in Australia and the Multi-Region Ethics Committee in New Zealand.

Participants in this trial will be unable to provide informed consent for themselves to participate in the study at the time of enrolment. A delayed consent model has been approved by the responsible ethics committees, which means a patient’s legal surrogate, relative/friend or whanau member will be approached for consent to participate in the study. Following consent from a patient’s legal surrogate, relative/friend or whanau member, the patient will be approached to give consent to continue in the trial if they recover the ability to do so and the timing is appropriate.

### Sample size and power

Using two published RCTs on nutrition therapy in ANZ critically ill patients, we estimated that the usual care group would receive an average of 1,400 kcal/day. We aim to deliver an additional 420 kcal/day (using a standard deviation of 600 kcal/day) to the intervention group, which is a 30 % relative increase in energy delivery and requires a sample size of 100 patients (80 % power, significance 0.05).

This sample size will also provide baseline rates of other key secondary outcomes which could be used in the future to inform sample size estimations for larger RCTs assessing clinical outcomes.

### Statistical analysis plan

Statisticians at the Australian and New Zealand Intensive Care Research Centre (ANZIC-RC) will perform statistical analysis using the intention-to-treat principle. All data will initially be assessed for normality and will be log-transformed as appropriate. Baseline variables and single measure outcomes will be compared using chi-square tests for equal proportion (or Fisher’s exact tests if numbers are small), Student’s *t*-test for normally distributed outcomes and Wilcoxon rank-sum tests otherwise. Continuously normally distributed repeated measure outcomes will be compared between groups using longitudinal mixed modelling fitting main effects for treatment and time with an interaction between treatment and time to determine if groups behave differently over time. Sensitivity analysis accounting for site, known covariates and baseline imbalances will also be performed for all outcomes, using logistic regression for binomial outcomes and mixed linear or non-linear modelling for continuous outcomes. Analysis will be performed using SAS version 9.4 (SAS Institute Inc., Cary, NC, USA), and a two-sided *p*-value of 0.05 will be considered statistically significant.

### Data and safety monitoring

Given the size of the trial, there are no planned interim analyses, and there is no dedicated data safety monitoring board. Safety will be monitored by reported adverse events and SAEs and reviewed by the study management committee (listed in Appendix 1) and Baxter Healthcare Corporation. All study sites will have an initial monitoring visit conducted by the project manager after two to five patients have been recruited. At this site visit, one intervention and one usual care arm patient will have 100 % source data verification at this visit; all other patients monitored at the visit will have consent procedures and eligibility criteria checked. Furthermore, intervention patients monitored at this initial visit will also have intervention delivery reviewed for adherence to the study protocol. Additional monitoring visits will be completed based on recruitment rates per site and any identified issues which need review after the initial monitoring visit.

The project manager will conduct remote monitoring of data completeness via the study website, and any data queries will be sent to the site for review.

## Discussion

Nutrition is a commonly used therapy in the ICU. It is relatively inexpensive compared to other treatments and, if used correctly, may positively affect clinical and functional outcomes, although this remains to be definitively determined. Large-scale RCTs to date have failed to deliver EN to meet estimated energy requirements, or have delivered nutrition in a population or manner that makes the evidence difficult to translate into clinical practice. This study aims to determine if a supplemental PN strategy will safely deliver close to 100 % of energy requirements compared to usual care, identify a patient population who may benefit most and minimise the risks of overfeeding. This information will assist in the development of future studies to provide definitive answers on the role of energy intake in critical illness.

### Trial status

The trial commenced recruitment on 17 February 2014. Final recruitment is expected to be achieved in late 2015 with 6 month outcomes available by early 2016.

## Appendix 1

Management committee of the Supplemental Parenteral Nutrition in Critically Ill Patients Phase II Randomised Controlled Trial

Shay McGuinness, Emma Ridley, Andrew Davies, Rachael Parke, David (Jamie) Cooper, Lyn Gillanders, Colin McArthur, Neil Orford, Owen Roodenburg.

## Additional file

Additional file 1:
**Detailed product information for the interventional product.** (DOCX 14 kb)

## Abbreviations

ANZ: Australia and New Zealand
ANZIC-RC: Australian and New Zealand Intensive Care Research Centre
BMI: body mass index
CBW: calculated body weight
CI: confidence interval
ECMO: extracorporeal membrane therapy
EN: enteral nutrition
EQ-5D-5 L: EuroQol 5 dimension 5 level
FiO_2_: fraction of inspired oxygen
GIT: gastrointestinal tract
HRQOL: health-related quality of life
ICU: intensive care unit
kcal: kilocalorie
kg: kilogram
mcg: microgram
min: minute
mmHg: millimetre of mercury
mmol: millimole
PaO_2_: partial pressure of oxygen
PN: parenteral nutrition
RCT: randomised controlled trial
RRT: renal replacement therapy
SAE: serious adverse event
