# Supplementary material for: Supplemental parenteral nutrition in critically ill patients: a study protocol for a phase II randomised controlled trial
Source: Trials. 2015 Dec 24;16:587. doi: 10.1186/s13063-015-1118-y (PMC4690293; doi:10.1186/s13063-015-1118-y)
Supplement: Additional file 1: — Detailed product information for the interventional product. (DOCX 14 kb) [file 13063_2015_1118_MOESM1_ESM.docx]

Additional file 1: Product information for Olimel N9-840E/Triomel 9 with electrolytes and additions

| **Contents** | **Compounded Ready To Use Parenteral Nutrition (per 1500ml bag)** |
| --- | --- |
| Total nitrogen (g) | 13.5 |
| Amino acid (g) | 85.4 |
| Glucose (g) (Hydrous) | 181.5  (equal to Anhydrous 165 g labelled on compounded bag) |
| Lipid as ClinOleic (g) | 60 |
| Total energy (kcal) | 1600 |
| Non protein energy (kcal) | 1260 |
| Glucose energy (kcal) | 660 |
| Lipid energy (kcal) | 600 |
| Sodium (mmol) | 52.5 (New Zealand)  54 (Australia - includes 1.5 mmol from Sodium Ascorbate) |
| Potassium (mmol) | 45 |
| Magnesium (mmol) | 6.0 |
| Calcium (mmol) | 5.3 |
| Phosphate (mmol) | 22.5 |
| Acetate (mmol) | 80 |
| Chloride (mmol) | 68 |
| Osmolarity (mOsm/L) | 1310 |
| **Additions per bag of PN** | |
| Baxter’s Multiple Trace Elements with Iron (mcg) | Per ml (note 10ml is added to each parenteral nutrition bag) |
| Zinc | 650 |
| Copper | 130 |
| Manganese | 27 |
| Chromium | 1 |
| Selenium | 3.2 |
| Iodide | 13 |
| Molybdenum | 1.9 |
| Iron | 120 |
| Ascorbate (Vitamin C) for stability (mg per bag)  Sodium Ascorbate in Australia and Ascorbate acid in NZ | 300 |
| Cernevit (ml per bag) | 5 |
